# What Did We Learn About Fracture Pain from Animal Models?

**DOI:** 10.2147/JPR.S361826

**Published:** 2022-09-13

**Authors:** Andreea Radulescu, Fletcher A White, Chantal Chenu

**Affiliations:** 1 Royal Veterinary College, Department of Comparative Biomedical Sciences, London, NW1 OTU, UK; 2 Department of Anesthesia, Indiana University School of Medicine, Indianapolis, IN, USA; 3 Richard L. Roudebush Veterans Medical Center, Indianapolis, IN, USA

**Keywords:** pain, fracture, mouse, behaviours

## Abstract

Progress in bone fracture repair research has been made possible due to the development of reproducible models of fracture in rodents with more clinically relevant fracture fixation, where there is considerably better assessment of the factors that affect fracture healing and/or novel therapeutics. However, chronic or persistent pain is one of the worst, longest-lasting and most difficult symptoms to manage after fracture repair, and an ongoing challenge remains for animal welfare as limited information exists regarding pain scoring and management in these rodent fracture models. This failure of adequate pre-clinical pain assessment following osteotomy in the rodent population may not only subject the animal to severe pain states but may also affect the outcome of the bone healing study. Animal models to study pain were also mainly developed in rodents, and there is increasing validation of fracture and pain models to quantitatively evaluate fracture pain and to study the factors that generate and maintain fracture pain and develop new therapies for treating fracture pain. This review aims to discuss the different animal models for fracture pain research and characterize what can be learned from using animal models of fracture regarding behavioral pain states and new molecular targets for future management of these behaviors.

## Introduction

Bone fractures from trauma and osteoporosis are a very common clinical problem affecting 3.6% of the UK population every year^1^ and this incidence is similar worldwide.^2^,^3^ Fracture occurrence is increasing due to the aging population and the incidence of diabetes, which largely contributes to these fragility fractures.^4^ While bone fractures typically heal well with appropriate treatment, a feared complication is the possibility of pain. Complex Regional Pain Syndrome (CRPS) can also be triggered by fracture injury and is a serious complication associated with severe pain.^5^

Three stages of pain are associated with bone fracture: acute, sub-acute, and chronic pain. Acute pain occurs immediately after the fracture, but typically dissipates with time, while the manifestation of sub-acute pain can continue for weeks.^6^ Sub-acute pain is thought to be associated with soft tissue injury and muscle weakness. Chronic pain is evident after healing is complete and may be associated with several tissue elements including peripheral nerve damage or the development of scar tissue.

Chronic or persistent neuropathic pain (NP) is one of the worst, long-lasting and most difficult symptoms to manage after fracture repair. It is likely that some of the pathophysiologic mechanisms leading to NP propagate early after injury, leading to the opportunity for early interventions. NP associated with bone fracture originates from a lesion affecting the peripheral nervous system and may be associated with abnormal sensations called dysesthesia or from normally non-painful stimuli (allodynia). The condition may have continuous and/or episodic (paroxysmal) components, with the latter resembling stabbing pain or electric shocks. Conditions associated with bone fracture-associated NP (BFNP) include traction neuropathy, nerve compression from soft tissue oedema, bone fragment, hardware and hematoma.

Much of the advances in bone fracture research are due to the disposal of animal models, particularly rodents, because they are easy to use, cheap and fracture healing occurs in a few weeks. The availability of genetically modified mice has contributed to favour the use of this species, although the bones are very small for fracture fixation. This has led to the development of reproducible models of fracture with more clinically relevant fracture fixation where there is considerably better assessment of the factors that affect fracture healing and/or novel therapeutics.^7^ An ongoing challenge remains for animal welfare as limited information exists regarding pain scoring and management in rodent fracture models.^8^ This failure of adequate pre-clinical pain assessment following osteotomy in the rodent population may not only subject the animal to severe pain states but may also affect the outcome of the bone healing study.^9^

Interestingly, the animal models developed to study pain were also mainly rodents and various models of somatic, inflammatory and neuropathic pain were described.^10^ It is however very difficult to measure pain in rodents and most of the measurements of pain are indirect assessing naturalistic behaviours and nociceptive reflexes. Including these pain measurements in animal models of fracture may lead to both better management after fracture^11^ and understanding of the mechanisms contributing to the mechanistic transition from acute and subacute to chronic pain states.

This review aims to discuss the different animal models for fracture pain research and characterize what can be learned from using animal models of fracture regarding behavioral pain states and new molecular targets for future management of these behaviors.

## Animal Models Mostly Used to Study Fracture Pain

### Choosing a Model

Building the most suitable animal model of fracture is based on the availability of species, age, sex, choice of the bone, the type of fracture and the mechanism of bone repair. A robust and consistently reproducible model is required, but a standardized model has been a challenge to date as different disease modelling systems have different needs and study design. In addition to reproducibility, relevance to the clinical case needs to be considered as the model should closely recapitulate the most critical aspects of the clinical scenario. When choosing a model for skeletal pain research, a high resemblance between the human and the animal model of choice in terms of skeletal biology and nociception/nervous system is required.

Bone repair models have been covered in depth in previous reviews.^12–16^ Many variables are in play: 1) the type of fracture repair, eg normal/delayed union/non-union; 2) the aetiology of fracture, eg osteotomy creating a clean fracture/impact, device creating a more traumatic fracture scenario but less replicable; 3) open versus closed fracture; 4) choice of species and ultimately 5) the fixation method, ie using a pin/external plate/using parallel bone technique, the latter being less reliable, due to potential deformities/differences in bone repair.

Large animals, such as pigs, are preferred as models of human fracture due to the presence of Haversian systems. In contrast, molecular and genetics tools make small animal models such as mice more versatile and suitable. A downside to the bone fracture models utilizing rodents is that bones continue to grow slowly with age and there is a lack of osteonal remodeling in cortical bone.^17^ Sexual dimorphism and age also need to be taken into account when modelling fracture. Female bone repair is slower than males due to reduced mesenchymal stem cell numbers.^18^ Inter-strain differences in murine models also influence fracture reproducibility in addition to other physiological aspects of the mice such as age and weight.^19^ Therefore, it is important to be aware of strain-specific characteristics that may bring limitations to the model system and hinder variability within results in fracture healing studies.

The use of rodent models is accompanied by the complication of size. Fracture healing in long bones can easily be studied using the tibia or the femur due to the relatively short time of complete healing of about 4 weeks in mice^20^ and 5 to 6 weeks in rats,^21^,^22^ allowing us to study all stages of healing. However, short bone fractures present a challenging difficulty, due to fixing mechanisms. For instance, in osteoporosis, the most commonly occurring fractures are of the wrist, vertebrae and femoral neck. Whereas models of diaphyseal and metaphyseal fractures^23^ have been proposed, vertebral fractures are still difficult to model.

### Models of Fracture Pain

While preclinical models using rodents have yielded insights that appear relevant to mechanisms responsible for bone fracture pain, they are limited in scope. The vast majority of studies examining pain behaviours after fracture were conducted in the Einhorn closed fracture model.^24^,^25^ Specifically, most studies are based on mice or rats using a closed mid-diaphyseal fracture of the left femur or tibia 14 days or 21 days after pin placement.^26–29^ Alternatively, some groups have utilized a rat or mouse tibial bone which is fractured using pliers with an adjustable stop followed by hindlimb casting. Following four to six weeks of immobilization, the cast is removed.^30–36^ This later model was mainly used as a model of complex regional pain syndrome (CRPS), a condition that develops after a range of injuries including fractures. Fracture-induced pain in animal models has not been studied thoroughly, but most studies of pain behaviours following fracture have been characterised in these models of closed fracture and CRPS.

Formerly known as reflex sympathetic dystrophy syndrome, CRPS is a condition that commonly affects a single limb and is accompanied by excruciating pain, changes in skin temperature and swelling or a combination of those symptoms. However, the model is not directly comparable to non-pathological fracture-induced pain due to the immobilisation of the limb, which induces physiological changes such as oxidative stress, vascular and inflammatory alterations that contribute to the pain phenotype observed which are not directly attributable to the fracture itself.^37^ Most studies of fracture pain used mice, but the rat model of closed femur fracture model has the advantage of a more accessible central nervous system for electrophysiology studies of pain compared to the mouse.^38^

### Fracture Rodent Model Complications That Affect Pain

Factors that influence fracture pain, in addition to the type and care of the fracture, depend on the fixation methods. A stainless steel intramedullary pin can be used to stabilize a fracture. This method is very simple and can be achieved by inserting a syringe needle in the intercondylar notch allowing the study of bone healing. It has been previously used for the molecular characterization of fracture callus in aged and osteoporotic mice,^39^,^40^ the effects of chronic inflammation on fracture healing^41^ and the effect of brain injury on bone healing.^42^ However, this is less suitable for the study of pain as the procedure itself could lead to a source of pain and confounding results due to damage to the intramedullary cavity. Additionally, the lack of rotational and axial stabilities in this model disadvantages histological analysis due largely to the disruption created by the removal of the pin following euthanasia. These complications could arise in spite of refinements of this method such as the use of locking nail, which improves stability while maintaining the simplicity, the low cost and minimal invasiveness of this technique.

To bypass the potential complications and pitfalls associated with this model, transverse osteotomy models stabilised with an external fixator have been developed. The osteotomy can be fixed with an external composite material plate with four pins, which allows the fixation of the bone in a reproducible manner, with little variation in bone healing positioning. The degree of elasticity of this model allows for endochondral and intramembranous ossification, which is the closest to the physiological healing of a fracture. The mechanism allows for different degrees of flexibility up to complete stability fixation of the osteotomy.^43–45^ The advantage over the internal fixation is that the external plate allows for a closed fracture surgery, while the implant does not touch the bone but offers the same level of stability.^45^ This model was recently used to study pain behaviours after fracture.^46^ In this model, the pain-like phenotype was maintained 6 weeks after fracture surgery.

The internal fixation model functions similarly to the external fixation, except it uses a rigid internal plate, which allows for a closed surgery^47^ and metaphyseal fixation^48^ that closely replicates the clinical fracture localisation in the femur. This is a fixed model, not allowing for modifications in the distance between the two bone ends, which may be necessary in some non-union fracture studies or even in secondary fracture studies. In spite of its relatively invasive nature and complex surgical technique, this model has the advantage of no medullary cavity damage and allows for a closer replication of the clinical scenario of a hip fixator.^49^ The model with internal fixation was however not employed to study fracture pain.

These models of fracture pain have been very useful to characterise acute and subacute pain after fracture. Pain can persist when a fracture does not heal and central sensitization can amplify the perception and the severity of pain. This has been less studied after fractures. In most studies, fracture healing occurs and mice and rats are kept for a few weeks after fracture, making the study of chronic pain after fracture difficult.

## Available Methods to Measure Fracture Pain in Rodents

In humans, pain reports are validated by questionnaires, qualitative sensory testing and pain scale methodology, which cannot be extended to animals. Most often the criteria used are subjective and based on a general clinical impression of the animal’s appearance and demeanour. However, using mouse models of fracture, we learned which techniques are useful to examine pain and what can we use them for.^29^ Many of the methods quantify “pain like” behaviours in rodents via testing responses to an external stimulus or measuring spontaneous behaviour.^8^ However, these pain measurements are likely indirect assessments of pain due to the lack of direct stimulation of bone nociceptive nerve fibres. Moreover, many studies investigating fracture pain also utilize changes in weight distribution and activity levels.^27^,^38^ Adequate validation of bone fracture pain states subsequently necessitates the use of several independent measures and ascertains correlations between them.

Standard tests that are used are studying both evoked and non-evoked naturalistic behaviours.

### Evoked-Pain Behaviours

Evoked pain behaviours have been thoroughly characterised and reviewed before^50^ and they include von Frey,^51^,^52^ cold plate,^53–55^ static weight-bearing,^56^,^57^ CatWalk^58^ and Laboras.^59^ Mechanical and thermal hyperalgesia were reported to be maintained up to 6 weeks after fracture surgery.^46^ These tests require interaction between the animal and the experimenter and can be subjective. When measuring evoked behaviours, consistency of environment, experimenter and other controllable variables is important,^50^ in addition to proper randomisation of animals.^60^

### Spontaneous Pain

Non-evoked behaviours are used as a way to non-specifically measure changed behaviour in rodents and are a measure of spontaneous pain. It has been shown that naturalistic behaviour measurements in rodents can capture behaviours related to pain, such as changes in activity and rearing^61^ using monitoring systems that reduce observer bias. Handling of rodents has a significant effect on their behaviours^62^ and naturalistic behaviours are less affected when observed by monitoring systems. Guarding and flinching are commonly used measurements of an ongoing or spontaneous fracture-induced bone pain.^27^ Observation periods are often two minutes during which time the number or duration of behaviour is counted.^63^ Jimenez-Andrade et al^27^ showed that guarding, flinching and weight-bearing had returned to baseline levels in both young and old rats 14 days after fracture, or close to baseline in adult rats. It is therefore questionable whether these naturalistic behaviours can capture the later phases of fracture-induced pain. The burrowing test is increasingly used to quantify spontaneous behaviour and represents a valuable test for assessing skeletal pain.^64^ Combinations of these methods rather than individual pain assessments improve reliability. Conditional place preference test is not routinely used to assess spontaneous pain but is a valuable method for analgesic drug assessment against chronic pain.^65^

## What Did We Learn About Fracture Pain from Animal Models

The study of pain to identify the mechanisms underlying it and the treatments to alleviate it has relied extensively on animal models. Early pain work was often performed on companion animals, but overtime, the research was conducted in mice and rats due to the wide variety of genetically modified strains that became available and ethical considerations. Using these pre-clinical models of fracture pain in rodents described above, we learned more on acute pain after fracture, changes in bone innervation, nociception processes and pharmacological treatments.

### Identification and Molecular Phenotype of Nerves in the Bone

Immunocytochemistry on bone sections taken from animals as well as animal models of fracture and pain have largely contributed to our knowledge of bone innervation and its changes with age and skeletal diseases. Since earlier work demonstrated that bone is densely innervated,^66^,^67^ several studies have described the phenotype of nerve fibres in bone.^68^ Both autonomic and sensory nerve fibres were identified in bone.^69^ Sympathetic adrenergic neurons expressing tyrosine hydroxylase are abundant in bone but there is still no evidence for parasympathetic innervation, although cholinergic fibres were identified in the periosteum, mainly derived from postganglionic sympathetic neurons.^69^ Sensory nerve fibres in the bone were most described. They are small unmyelinated C fibres and myelinated Aδ axons that are mainly peptide-rich, expressing neuropeptides such as Calcitonin Gene Related Peptide (CGRP) and substance P (SP). Most express neurotrophic tyrosine kinase receptor 1 (TrkA) a receptor for nerve growth factor (NGF) and this differentiates them from nerve fibres expressed in the skin.^70^ These nerves play a role in fracture healing as repair is affected by denervation^27^,^71^,^72^ and neuropeptides expressed in bone.^73^ Complete denervation of bone tissue is known to impair fracture healing, while the presence of primary afferent sensory innervation contributes to fracture healing.^74^ In contrast, we showed that treatment with propranolol, an antagonist of the beta-adrenergic system and associated sympathetic fibres, fails to affect fracture healing and callus strength.^75^ It also appears that fracture repair requires TrkA signaling or calcitonin-gene related peptide release by skeletal sensory nerves.^76^,^77^

Peripheral nerves associated with bone also change with age and diseases. Although the findings from several studies suggest that the density of bone innervation does not change with age,^78^,^79^ there does appear to be a correlation with the amount of bone present.^80^,^81^ In contrast, ectopic nerve sprouting occurs after fractures^82^ but does not appear to exist following successful bone union. Moreover, a mouse model of diaphyseal fracture with an external fixator fails to exhibit enhanced number and density of nerve fibres in bone when assessed 6 weeks after fracture.^46^ In contrast, excessive nerve sprouting is maintained in non-healed fractures and may contribute to chronic skeletal pain.^83^

### Nociception After Fracture

Due to the limited access to bone biopsies in humans, the understanding of the mechanisms leading to nociception after fractures is largely limited to the use of animal models and techniques, such as immunocytochemistry, gene expression analyses, imaging, anterograde tracing and nerve conduction studies.^84^

After fractures, several mechanisms have been proposed to induce skeletal pain and they can interact with each other. The distortion of the rich-innervated periosteum during injury precipitates a series of events immediately after fracture. The mechanosensitive sensory nerve fibres A-delta and C are rapidly activated and movement and loading of the fracture bone becomes painful.^24^ Activation of small diameter unmyelinated C nerve fibres is likely to generate the slow burning bone pain. There is an immediate release of various signalling molecules neurotransmitters, neuropeptides, cytokines and neurotrophins from cells or peripheral nerve processes at the fracture site.^73^ These molecules contribute to the stimulation and sensitization of nociceptors. They can also induce ectopic nerve sprouting as demonstrated for NGF.^24^ Many inflammatory mediators discharged after fractures contribute to fracture pain sequela. This inflammatory phase together with bone resorption activity necessary to remove bone debris create a state of fracture tissues acidosis which can effectively activate acid-sensing channels expressed on nociceptors such as acid-sensing ion channels (ASICs) and transient receptor potential vanilloid (TRPV) channels, further contributing to bone pain.^85–88^

The advantage of mouse models is that it allows the use of genetically modified mice. This possibility has also largely contributed to our knowledge of pain after fracture. The role of kinins (Bradykinin) in inflammation and fracture pain, for example, was demonstrated using mice that are deficient in kinin receptors.^89^ These receptors are highly expressed at the fracture site and mice deficient in B1 and B2 receptors demonstrate a reduction in post-fracture pain sensitivity. Both receptors were shown to contribute to fracture pain via COX-1/COX-2 signalling.

A number of small animal models of fractures also allows the examination of different cell interactions at the fracture site. For example, Li et al demonstrated that neurotrophic factors such as brain-derived neurotrophic factor (BDNF) or neuropeptides such as Substance P (SP) released from afferent C-fibers can stimulate bone formation during fracture healing.^90^ Glia cells also contribute to the healing process by myelination and pain signals transmission.^91^ Following bone fracture, mechanical injury to nerve fibres may also occur, generating neuropathic pain states.^92^

Animal models also offer a good description of neurochemistry and anatomy. In bone, retrograde labelled sensory experiments in rats have shown anatomical connections between sensory neurons innervated the periosteum and the medullary cavity and ipsilateral DRG spanning lumbar regions L1 to L6.^93^ As it is thought that nociceptive input can induce a prolonged increase in excitability and synaptic efficacy of neurons, nerve fibres sensitization following fracture may serve to amplify pain perception and severity.^94^ These attributes are, however, poorly studied in animal models of fracture.

### Pathophysiological Neurochemical Changes

Animal models should provide a basis for the pathomechanisms of human diseases. As in humans, it was shown that fracture-induced pain behaviours peak at 2 days post fracture in rodents and decline with soft callus formation.^95^ Other studies have, however, shown longer pain behaviours after fracture. Using a mouse model of diaphyseal fracture with external fixator, we showed that the fracture group had  persistent mechanical and thermal hypersensitivities six weeks after fracture, indicative of a chronic pain-like phenotype.^46^ Animal models of fracture make it easier to correlate these pain behaviours with the healing of fracture, bone remodelling and neurochemical changes in the DRGs or spinal cord.

### Comparison of Drugs to Alleviate Fracture Pain

Rodent fracture models, when standardised, allow pain treatment comparison and can identify potent and effective drugs that have the potential to be effective in clinic. While many studies have examined the effects of drugs on fracture healing, more recently, these fracture models also allowed the examination of several therapies on fracture-associated pain such as the anti-nerve growth factor (anti-NGF) therapy, opioids and NSAIDs. The most studied is the anti-NGF and several studies using a rat tibia fracture model of complex regional pain syndrome have demonstrated that anti-NGF antibodies elicit strong anti-nociceptive effects.^26^,^36^ More recently, anti-NGF administered before and weekly after surgery alleviated fracture-induced mechanical nociception in a femoral fracture model with external fixation.^46^

The use of opioids raises many concerns related to dose-dependent increases in long-term pain and major side effects. Morphine was shown to reduce spontaneous pain and increase weight bearing 7 days post fracture in rats.^38^ Buprenorphine is commonly used as an analgesic treatment for post-operative pain in small animals.^96^ However, emerging evidence suggests that opioid use immediately after trauma worsens outcomes as it impairs nociceptive recovery and alters postfracture expression levels of several genes.^97^

The use of non-steroidal anti-inflammatory drugs (NSAIDs) is common in a number of trauma-induced inflammatory pain conditions in both pre-clinical and clinical studies and has been shown to lower pain scores and reports. However, the use of NSAIDs in bone healing is limited as there are some fears that they may affect bone health and repair.^98^ The beneficial effects of bisphosphonates on the reduction of skeletal pain were mainly studied in the context of bone cancer wherein there was an apparent ability to inhibit pain but they produced bone loss in a rat tibia fracture model of complex regional pain syndrome.^32^ Anti-sclerostin therapy also prevents painful-age related fractures,^99^ although it is still not known if it can directly affect nociception.

## Limitations of Fracture Pain Studies in Animals

Although laboratory animals have been extensively used to model human diseases, they have several disadvantages when studying fracture pain. First, pain is often associated with musculoskeletal diseases that are frequent in the elderly population. While life expectancy in the elderly population can reach over 80 years, mice which are often used to study skeletal pain only live for two years. With age, there are different changes in the brain and in the somatosensory system that are very different in humans and mice.^100^ Second, patients are not similar, and there are variations in their individual response to pain due to different mechanisms and sensory profiles.^101^ This variation is impossible to model in mice which have been highly selected and bred. The same strains of mice are used extensively, for example, the strain C57BL/6J, and utilised as a single representative of the species, limiting the interpretation of the data and the conclusions drawn.^102^

### Differences in Physiological Mechanisms Between Humans and Animals

Many aspects need to be considered when using animal models to study fracture pain. One major physiological difference between animals and humans is related to their size.^7^ There are distinct interspecies differences with regard to bone composition, density and mechanical competence.^103^,^104^ Furthermore, bone remodelling was shown to be partly controlled by the brain^105^ and we cannot exclude that the size of the brain may affect the central control of bone mass. It may also shape pain processing, although it was shown that animals share similar mechanisms of pain detection than humans and have similar areas of the brain involved in processing pain. Not many controlled studies have compared mechanisms of acute and chronic pain in mammal studies considering size, sex and age. Previous literature has found that studying pain neurophysiology can be very different depending on the animal models. Pain research in larger mammals is rare due to welfare issues, but pain is highly studied in dogs, for example, for bone cancer pain, using a scale for guessing pain severity. Dogs are interesting as it was shown that they approximate human properties the best in terms of bone quantity and quality.^103^ Similarities and differences in nociceptors between human and rodents have shown molecular and physiological differences between pain pathways, illustrating some difficulties in translating some of the findings in mice into humans.^106^ Efficacy of pain treatments on fracture healing also illustrate differences between animal models. This was shown, for example, with the effects of NSAIDs on fracture healing, which were effective on small animals but not on large animals, such as dogs and goats.^98^

### Genetics

It is not easy to study pain in humans as it is influenced by many factors including genetics. Many inter-individual differences in pain are reported that could be explained by changes in DNA, gene expression and epigenetics.^107^ Pain sensitivity is also heritable in rodent models of pain, and this was particularly shown by selective breeding of mice. Mice are often used to study pain, mainly due to their inexpensive maintenance, easy breeding, the fact that there are many strains available but also due to the advantage of using transgenic mice available in this species. However, the use of knockout mice means that most studies examining pain are comparing genotypes which differ greatly from each other on a large variety of pain-related traits.^108^ The background strain has a significant influence on nociception, and the pain response can be very different to other inbred and outbred mouse strains.

### Pain Perception and Psychology

Pain is a subjective experience and involves both emotional and social experiences.^109^,^110^ Assessing pain in humans then requires performing tools that analyse all aspects of pain experience such as the Mc Gill pain questionnaire or the Brief pain inventory. Humans can define the highly complex nature of their pain experience after fractures.^63^ Similarly, pain in animals has been defined as “an aversive sensory experience caused by actual or potential injury that elicits protective motor and vegetative reactions, results in learned avoidance behaviour, and may modify species specific behaviour including social behaviour”.^111^ Defining reliable pain scales in animals is difficult due to the large variations in pain responses. Most pain assessments in animal models do not measure cognitive appraisal or integrate the animal’s emotional response.^112^ Clinical fracture pain is a perception that is influenced by psychological and experiential factors. Interestingly, humans have been shown to be the best species to heal due to their psychology,^113^ suggesting that recovery after fracture may be sensitive to social cues. It is not possible to fully recapitulate this emotion and psychology in an animal fracture model.^114^ Emotional experience is what distinguishes true pain from nociception, which is the sensory nervous system’s process of encoding noxious stimuli and processing signals to the brain via the spinal cord. This experiential component is likely to vary from animals to humans and between animals of the same species. Pain in animals is likely to be adaptive in an evolutionary sense, animals having evolved methods of suppressing responses to pain to enhance survival.^115^ This phenomenon is likely to exist in prey animals such as rodents.^111^ Pathophysiologic stress is also very important when assessing pain in both animals and humans. This stress is displayed differently between species. In humans, it can lead to depression and anxiety altering the perception of pain. In laboratory animals, distress can manifest in abnormal respiration, reduced grooming, piloerection, hunching, absence of alertness. The investigation of placebo effects in animal pain research is difficult because animals cannot report their pain and rate their experience. Placebo analgesia in rodent pain research is, however, increasingly used and there is hope for new developments.^116^

### Assessing Long-Term Chronic Pain

Most studies of pain in animals have investigated acute pain states such as observed post-surgery. Persistent pain is common after fracture surgery in humans^117^ but assessing chronic pain which outlasts bone healing is more difficult to perform in animals, firstly due to the paucity of chronic pain models and the lack of reliable tools. There is limited information of chronic pain in free-living animals, which may be due to the poor survival of animals with chronic pain, the lack of observations and behaviours assessments. Central sensitization was shown in rodent studies, but behaviours associated with this pain are difficult to assess and may be mainly related to avoidance of pain exacerbation.^118^ Nociceptive withdrawal reflex has been suggested as a useful biomarker for central sensitization in rodents.^119^ In addition, due to ethical considerations, it is increasingly difficult to keep animals for long-term studies. Most fracture pain experiments in rodents are performed in a few weeks and do not examine the long-term effects of pain.

### Imaging Technology

Many advances in our understanding of pain in humans are the results of the development of functional neuroimaging techniques. They can measure changes in neuronal activity by assessing alterations in blood flow, oxygen and glucose metabolism, but also neurotransmitter uptake and binding to its receptor.^120^ Functional changes in the pre-clinical brain due to chronic pain have not been fully elucidated, as brain imaging techniques including functional magnetic resonance imaging and positron emission tomography (PET) require the use of anesthesia to suppress movement. However, some insights into possible reflection of pain states have recently been described for neuropathic pain imaging in awake animals and include enhanced neural activity in several brain regions.^121^

### Weaknesses Associated to Pain Measurements in Rodents

Most reported measurements of pain in rodents are made during the light phase, not taking into account the natural circadian rhythm of the animal as rodents are nocturnal. This is likely to impair pain assessments. Furthermore, the most commonly used endpoint in assessing skeletal pain in rodents is skin hypersensitivity assessed by von Frey. This does not reflect the clinical situation of a patient with skeletal pain, as skin hyperalgesia is rarely the major pain complaint but rather the spontaneous pain that arises from the bone under usage or localised in the immediate region around the fracture site.

## Conclusions

Because pain is one critical factor that controls bone loading required for recovery after fracture, developing appropriate models to study fracture pain is essential. While the choice of animal models to study fracture repair is vast, the study of pain in these models requires very reproducible models that are the least invasive, enabling the analysis of pain due to the osteotomy itself and not pin placements. To date, the models of choice are 1) the closed mid-diaphyseal femoral fracture 21 days after pin placement, which is less invasive and allows for the surgical pain to resolve before fracture; 2) the open femoral fracture stabilised with an external fixator which is standardised and very reproducible but more invasive. None of these models are, however, suitable to study the pain associated with osteoporotic fractures as the fracture is performed in the femoral diaphysis, a site with no osteoporosis, and other models need to be developed. To improve the efficacy of translation of scientific data collected from animal models of fracture pain into the production of clinically affective analgesic agents, we will also need to develop more behavioural animal models and ideally better spontaneous measurements of pain. Although animal models can be valuable to select new compounds with an improved profile, they may be less helpful when assessing novel mechanisms. If we want to make progress in our understanding of fracture pain, both human and animal studies are complementary.
